# The Nucleoside Diphosphate Kinase Gene *Nme3* Acts as Quantitative Trait Locus Promoting Non-Mendelian Inheritance

**DOI:** 10.1371/journal.pgen.1002567

**Published:** 2012-03-15

**Authors:** Hermann Bauer, Sabrina Schindler, Yves Charron, Jürgen Willert, Barica Kusecek, Bernhard G. Herrmann

**Affiliations:** 1 Department of Developmental Genetics, Max Planck Institute for Molecular Genetics, Berlin, Germany; 2 Department of Biology, Chemistry, and Pharmacy, Free University Berlin, Berlin, Germany; 3 Institute for Medical Genetics, Charité – University Medicine Berlin, Berlin, Germany; Institute of Molecular Genetics of the ASCR, v.v.i., Czech Republic

## Abstract

The *t*-haplotype, a variant form of the *t*-complex region on mouse chromosome 17, acts as selfish genetic element and is transmitted at high frequencies (>95%) from heterozygous (*t/+*) males to their offspring. This phenotype is termed transmission ratio distortion (TRD) and is caused by the interaction of the *t*-complex responder (*Tcr*) with several quantitative trait loci (QTL), the *t*-complex distorters (*Tcd1* to *Tcd4*), all located within the *t*-haplotype region. Current data suggest that the distorters collectively impair motility of all sperm derived from *t*/+ males; *t*-sperm is rescued by the responder, whereas +-sperm remains partially dysfunctional. Recently we have identified two distorters as regulators of RHO small G proteins. Here we show that the nucleoside diphosphate kinase gene *Nme3* acts as a QTL on TRD. Reduction of the *Nme3* dosage by gene targeting of the wild-type allele enhanced the transmission rate of the *t*-haplotype and phenocopied distorter function. Genetic and biochemical analysis showed that the *t*-allele of *Nme3* harbors a mutation (P89S) that compromises enzymatic activity of the protein and genetically acts as a hypomorph. Transgenic overexpression of the *Nme3 t*-allele reduced *t*-haplotype transmission, proving it to be a distorter. We propose that the NME3 protein interacts with RHO signaling cascades to impair sperm motility through hyperactivation of SMOK, the wild-type form of the responder. This deleterious effect of the distorters is counter-balanced by the responder, SMOK^Tcr^, a dominant-negative protein kinase exclusively expressed in *t*-sperm, thus permitting selfish behaviour and preferential transmission of the *t*-haplotype. In addition, the previously reported association of NME family members with RHO signaling in somatic cell motility and metastasis, in conjunction with our data involving RHO signaling in sperm motility, suggests a functional conservation between mechanisms for motility control in somatic cells and spermatozoa.

## Author Summary

Selfish genetic elements, which promote their own propagation and thereby violate Mendel's laws, have attracted much attention within the scientific community and by the public. The molecular principles underlying their exceptional behaviour are, in general, not well understood. A notable exception is the *t*-haplotype of the mouse, which was discovered in 1936 and has since been a paradigm for non-Mendelian inheritance in mammals. Recently we have revealed the molecular nature of several elements within this genetic region, including the *t*-complex responder and two *t*-complex distorters that interact to promote the high transmission rate of the *t*-haplotype from males (which carry this variant genetic region) to their offspring. Here we show that the nucleoside diphosphate kinase gene, *Nme3*, acts as distorter and thus contributes to the high transmission rate of the *t*-haplotype. We show that *Nme3* acts as quantitative trait locus and interacts with RHO signaling cascades involved in sperm motility control. Since human NME family members have been associated with somatic cell motility (which is also controlled by RHO signalling) and cancer cell metastasis, we propose a functional conservation between motility control in somatic and sperm cells.

## Introduction

In general, diploid organisms transmit homologous chromosomes at the Mendelian (equal) ratio to their offspring. However, several types of non-Mendelian inheritance have been described, and in mammals a prominent example is transmission ratio distortion (TRD) in the mouse, which is caused by the *t*-haplotype. The *t*-haplotype is a variant form of the *t*-complex, which maps to the centromere-proximal third of chromosome 17. According to evolutionary studies, this haplotype originated more than one million years ago and, due to four large inversions, has since evolved devoid of meiotic exchange with the wild-type *t*-complex [1]–[3]. The *t*-haplotype is transmitted at an abnormally high ratio from heterozygous (*t/+*) males to their offspring [4]. This selective advantage is due to superior swimming behaviour of *t*-haplotype sperm as compared to +-sperm derived from the same male [5], [6]. *t*-sperm does not, however, function superiorly to sperm derived from wild-type (+/+) males [7]. The *t*-haplotype rather encodes several *t*-complex-distorters (*Tcd*), which cumulatively affect sperm motility. This deleterious effect is rescued by the *t*-complex-responder (*Tcr*), but exclusively in *t*-haplotype carrying sperm. Thus, only sperm carrying the wild-type *t*-complex are affected.

The first evidence for how TRD is caused molecularly was obtained following the isolation of *Tcr*, which was found to encode a mutant, dominant-negative form of Sperm motility kinase 1 (*Smok1*), termed *Smok1^Tcr^* [8]. Expression of wild-type *Smok1* and of *Smok1^Tcr^* commences in haploid spermatids and, in contrast to other haploid expressed genes, neither their RNA nor their protein products are shared between haploid sperm cells, which are connected in a syncytium [9]. This exceptional behaviour provided a molecular explanation for the exclusive rescue of *t*-sperm from the deleterious effect of the distorters.

The molecular nature of SMOK^Tcr^ revealed that TRD is caused by alterations in a signaling pathway involved in sperm motility, and led to the identification of *Tcd* genes, which were postulated to act upstream of SMOK1 in this signaling pathway [8]. The first *Tcd* isolated was *Tcd1a*, which was identified as a hypermorph of *Tagap1*, a GTPase activating protein (GAP) and inhibitor of Rho small G proteins [10]. *Tcd2* was later shown to encode a hypermorph of *Fgd2* (Faciogenital dysplasia 2), a GDP/GTP exchange factor (GEF) and activator of the Rho protein CDC42 [11], [12]. These data established the involvement of Rho signaling in the control of sperm motility and in TRD. Rho G proteins are molecular switches that cycle between an active, GTP-bound, and an inactive, GDP-bound, state. GAPs enhance the hydrolysis of GTP, driving Rho small G proteins into the inactive state, while GEFs enhance the loading of small G proteins with GTP, thus promoting the active state.

Here we show that the nucleoside diphosphate kinase gene *Nme3* (protein expressed in non-metastatic cells 3; MGI acc. number 1930182, Ensembl gene ENSMUSG00000073435) acts as a quantitative trait locus in TRD. Group I nucleoside diphosphate kinases such as NME3 function to phosphorylate GDP to GTP, the activator molecule for small G proteins, providing a link between *Nme3* and the previously identified *Tcd* genes. We show that reduction in the *Nme3* gene dosage by gene targeting enhances the transmission rate of the *t*-haplotype, while transgenic over-expression of the *t*-allele reduces *t*-haplotype transmission. Genetic and biochemical data demonstrate that the *t*-allele of *Nme3* is a distorter and acts as hypomorph, in contrast to previously identified distorters. *Nme3* complements the family of G protein-related factors acting as QTLs in non-Mendelian inheritance.

## Results/Discussion

### *Nme3* Is Expressed in Testis and Is Altered in the *t*-Haplotype

**Figure 1:**
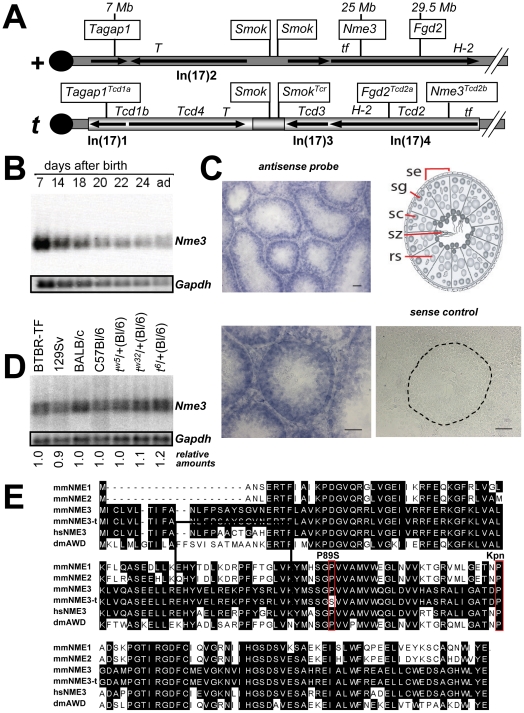
*Nme3* is a *Tcd2* candidate. (A) The position of *Nme3* in the wild-type *t*-complex (+) and genetic mapping on *t^w18^* localized it to the *Tcd2* region of the *t*-haplotype (*t*) (see Figure S1). Symbols of genes verified to be involved in TRD are boxed, and map positions relative to the wild-type chromosome 17 (in Mb) are indicated. Molecularly unknown *Tcd* loci are also listed along with Chr17 inversions and their relative orientations in both, the wild-type and *t*-haplotype chromosome, as well as crucial genetic markers (*T*, *tf*, *H-2*). (B) Northern blot analysis of *Nme3* transcripts in testes from consecutive post partum stages reflecting the first round of spermatogenesis (P7–P24), and in testes of adult mice (ad). (C) *In situ* hybridization analysis of *Nme3* on testis cryosections from an adult male. Expression is predominant in cells near the basal lamina (dotted line) representing diploid cell types. Schematic view of a seminiferous tubule. (Se) Sertoli cells; (SG) spermatogonia; (SC) spermatocytes; (RS) round spermatids; (SZ) spermatozoa. Scale bar: 50 µm. (D) Northern blot analysis of *Nme3* expression in different wild-type strains and *t*-haplotypes. Quantification of the signals with respect to C57Bl/6 did not reveal significant differences. (E) Amino acid sequence comparison of several NME proteins encoded by *Mus musculus* (mmNME1 to mmNME3), the *t*-haplotype (mmNME3-t), *Homo sapiens* (hsNME3), and *Drosophila melanogaster* (dmAWD); a conserved proline at position 89 is altered in mmNME3-t (P89S), and a nearby proline residue was described as killer-of-prune mutation (*K-pn*) in the abnormal-wing-disc (*awd*) gene of *D. melanogaster* when mutated to serine (red boxed).

The identification of the Rho small G protein regulators *Tagap1* and *Fgd2* as *t*-complex-distorters within the *t*-haplotype suggested that more genes involved in G protein signaling which may have a quantitative effect on *t*-haplotype transmission might be located within this chromosome segment. Therefore, we initiated a search for genes related to Rho signaling within this region of chromosome 17. We identified the gene *Nme3*, encoding a member of the nucleoside diphosphate kinase (NDK) family, at position 25 Mb from the centromere (Figure 1A). *Nme3* belongs to the group I *Nme*s (*Nme1*–*4*) which are all catalytically active and share significant sequence homology [13], [14].

In order to qualify as a *Tcd* candidate, a gene must be expressed in the testis and show variability between *t*- and wild-type alleles. Northern blot analysis showed expression of *Nme3* in testes from the earliest stage after birth tested (7 days) to the adult (Figure 1B). Using *in situ* hybridization on sections of adult testes, *Nme3* transcripts are detectable predominantly in early stages of spermatogenesis, while expression appears to be down-regulated in haploid cells (Figure 1C). However, the expression analysis of round spermatids using microarrays have shown that *Nme3* transcripts are also present in spermatids ([15], and EMBL-EBI: Gene Expression Atlas). Thus, *Nme3* transcripts apparently persist during spermiogenesis and allow translation of NME3 protein acting later in spermatozoa. In conclusion, *Nme3* was found to fulfill the first important criterion for a distorter.

Since the RNA expression level can be a good indicator of a QTL, as shown for the *t*-alleles of *Fgd2* and *Tagap1* [10], [11], we analyzed the expression of *Nme3* from the *t*-haplotype allele and compared it to the wild-type alleles from several mouse strains. The expression levels were found to be nearly identical between RNA derived from testes of C57BL/6 or other wild-type strains, and *t*-haplotype carrying strains (Figure 1D). *t^6^/*+ male testes showed marginally higher expression.

Since the analysis of RNA expression level revealed no significant variability between *t*- and wild-type alleles we examined sequence variation. We isolated cDNA clones by RT-PCR from testicular RNA from several wild-type and *t*-haplotype carrying strains and from a testis cDNA library prepared from *t^6^/t^w5^* males. In addition, we analyzed genomic fragments derived from several *t*-haplotypes. All sequence analyses detected a *t*-specific C to T transition in the coding sequence of *Nme3*, a missense mutation resulting in the change of proline to serine at position 89 (P89S; Figure 1E). This mutation was found in all *t*-haplotypes tested, which carry the *t*-form of the inversion In(17)4 (*t^w5^*, *t^w32^*, *t^w12^*, *t^6^*), but in none of the wild-type strains analyzed (C57BL/6, DBA/2, 129Sv, NMRI) (Figure 1E and data not shown). Therefore, this P89S mutation in *Nme3* distinguishes the *t*-allele from the wild-type allele. All group I NDK enzymes possess almost identical 3-D structures, and the mutation affects a highly conserved amino acid located between alpha-helix α2 and beta-sheet β3 of NME3 [13], [14], which may alter the function of the protein. A similar proline to serine exchange was described in the Killer-of-prune mutation (*awd^Kpn^*) of the abnormal-wing-disc (*awd*) gene of *D. melanogaster* (Figure 1E; [16]). The *awd^Kpn^* mutation was shown to decrease the nucleoside diphosphate kinase activity substantially with respect to the wild-type *awd* gene product [17].

The combined data identified the *t*-allele of *Nme3* as a distorter candidate.

According to its position on chromosome 17 it was not clear whether the *Nme3* gene is located within the *Tcd2* or *Tcd3* region. The proximal partial *t*-haplotype *t^w18^*, which extends into inversion In(17)4, carries *Tcd3*, but not *Tcd2*, which maps more distally [18], [19]. Therefore, analysis of *t^w18^* allows the assignment of *Nme3^t^* to either the *Tcd3* or *Tcd2* region. Genomic Southern blot analysis complemented by cDNA sequencing demonstrated that *t^w18^* carries the wild-type allele of *Nme3* and thus, *Nme3^t^* is a *Tcd2* candidate (Figure S1 and data not shown).

### A Knock-Out Allele of *Nme3* Phenocopies a Distorter

**Figure 2:**
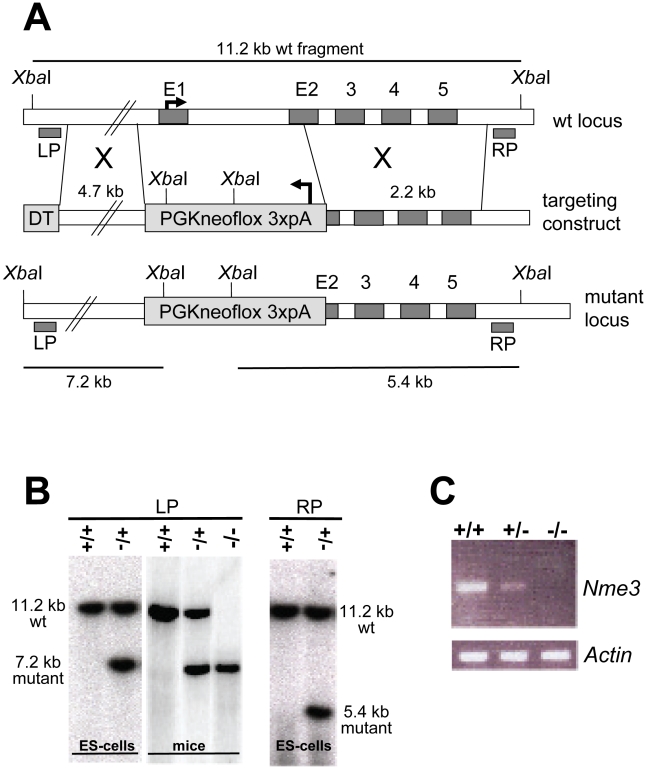
Targeted inactivation of the *Nme3* gene. (A) Gene targeting strategy: a neomycin selection cassette was used to replace the entire first and part of the second exon of *Nme3*. (B) Confirmation of correct homologous recombination by Southern blot analysis of *Xba*I digested genomic DNA derived from ES-cells or mice. (C) Loss of *Nme3* transcripts in homozygous knock-out mice (−/−) as determined by expression analysis using RT-PCR confirms that the targeted allele represents a null mutation. Genomic regions in (A) are not drawn to scale. Abbr.: LP, RP: left or right external probes; DT, diphteria toxin cassette for negative selection; wt, wild-type; +, wild-type allele; −, knock-out allele.

Distorter genes act as QTLs in the sense that up- or down-regulation of gene expression and/or activity has a quantitative effect on the phenotype; observed here as TRD. A proven method for testing a possible effect of gene dosage on TRD is to inactivate the wild-type allele by gene targeting, assay the transmission of a *t*-haplotype from males carrying the knock-out allele on the homologous chromosome, and compare it to control males which carry the wild-type allele. We targeted the *Nme3* gene in ES cells by replacing exon 1 and part of exon 2 with a Pgk-Neomycin resistance cassette, generating a null allele (Figure 2A). Successful integration of the targeting construct was verified by Southern blot analysis (Figure 2B). We introduced the targeted allele into the germ line and confirmed by RT-PCR that *Nme3* transcripts are lacking in the testes of homozygous-null males (Figure 2C). We then generated males carrying the targeted allele on the wild-type chromosome and the wild-type allele on either of the partial *t*-haplotypes *t^w18^* or *t^h49^*. Littermates carrying the wild-type allele on both chromosomes 17, in conjunction with *t^w18^* or *t^h49^*, served as controls. For each male we determined the number of offspring that inherited the *t*-haplotype. In both tests the *t*-haplotype was transmitted at a significantly higher rate (15% and 8% respectively) from males carrying one targeted allele compared to littermates homozygous for the wild-type allele (Table 1). Therefore, a reduction of the gene dosage by half significantly increased the transmission rate of the *t*-haplotype. These data demonstrate that *Nme3* acts as a QTL on *t*-haplotype inheritance.

**Table 1:** The reduction of the wild-type *Nme3* gene dosage and over-expresssion of the *Nme3 t*-allele have opposite effects on *t*-haplotype transmission.

		Offspring					
Genotype of male	Number of males	*t*	+	total	% *t*	χ^2^	P
*Nme3^tm5Bgh^*/+ *t^h49^*/+	7	167	308	475	35.2	6.721	0.0095
*Nme3^+^* ^/+^ *t^h49^*/+	7	117	317	434	27		
*Nme3^tm5Bgh^*/+ *t^w18^*/+	4	150	104	254	59.0	11.701	0.0006
*Nme3* ^+/+^ *t^w18^*/+	5	139	175	314	44.3		
*t^6^*/+ *Tg^t^/0*	8	587	72	659	89.0	5.337	0.0209
*t^6^*/+	8	646	50	696	92.9		

Abbr.: +, wild type; *Tg^t^*, *Tg(Nme3)H17bgh*.

### The *t*-Allele of *Nme3* Encodes a Hypomorph and Acts as Distorter

Although the genetic inheritance test proved the nature of *Nme3* as a QTL, it did not verify that the *Nme3 t*-allele acts as distorter gene. The latter requires that the *t*-allele itself alters the overall *Nme3* activity in sperm, and causes a statistically significant change in *t*-haplotype transmission. To determine the mechanisms through which this may happen we first assessed the enzymatic activity of NME3-P89S relative to wild-type NME3.

**Figure 3:**
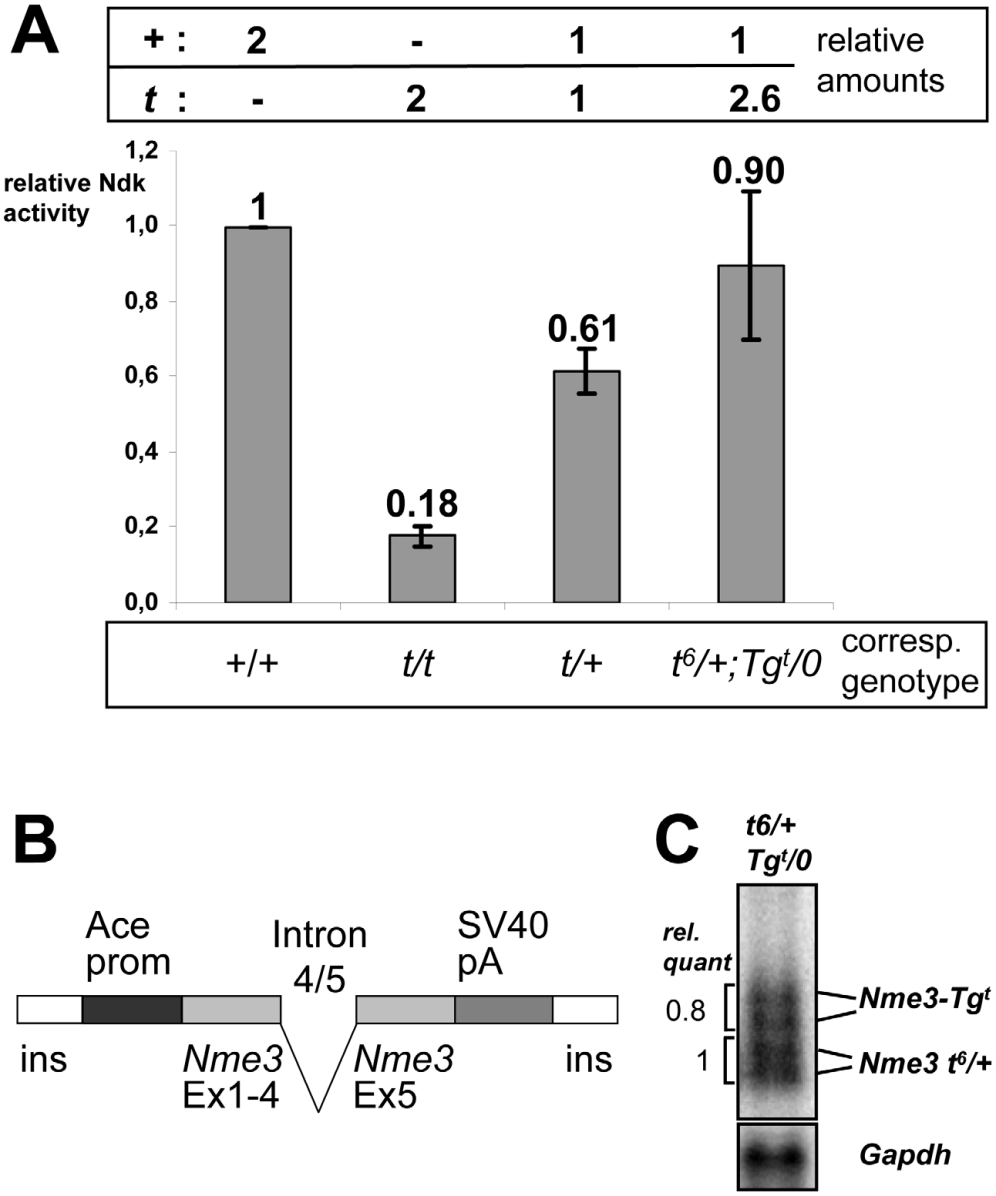
The *t*-allele of *Nme3* encodes a hypomorph. (A) Nucleoside diphosphate kinase activities of wild-type (+) and *t*-allele (*t*)-derived proteins or mixtures of both expressed *in vitro*. The 1∶1 mixture reflects the relative contribution of both alleles to NME3 activity in *t*/+ heterozygotes, the 1∶2.6 ratio of + and *t* alleles corresponds to the expression of + and *t* alleles measured in testes from *t^6^*/+;*Tg^t^*/0 males (B, C). The columns show the mean of three experiments with the standard deviation. (B) Schematic representation of the transgene construct *Tg(Nme3)H17bgh* (*Tg^t^*) generated for expression of the *Nme3 t*-allele during spermatogenesis. (C) Expression of the *Nme3 t*-allele from the transgene construct determined by Northern blot analysis of testis RNA derived from a *t^6^*/+;*Tg^t^*/0 male. Quantification of endogenous (*t^6^*, +) and transgene (*Tg^t^*)-derived transcripts revealed a ratio of 1∶0.8. Assuming that the *t^6^* allele contributes 50% of the endogenous *Nme3* transcripts, the overall ratio of + to *t* derived *Nme3* transcripts expressed in a *t^6^*/+;*Tg^t^*/0 male is therefore 1∶2.6. Abbr.: Ace-prom, angiotensin converting enzyme promoter; SV40pA, Simian virus 40 polyadenylation signal fragment; ins, chicken beta globin insulator [33]; Ex, exon; Ndk, nucleoside diphosphate kinase.

We produced the enzyme *in vitro* using a coupled transcription/translation system in rabbit reticulocyte lysate, purified the complexes, and measured the activity in an enzymatic assay. NME3-P89S showed strongly reduced enzymatic activity compared to wild-type (129Sv) NME3 protein (18% of wt activity; Figure 3A).

It is important to note that nucleoside diphosphate kinases function as hexamers. Therefore, *in vivo* the *t*-encoded NME3 monomers may form mixed hexamers with the wild-type protein, and in mixed complexes NME3-P89S might function as dominant-negative protein interfering with the function of the wild-type protein. Alternatively, it might form semi-functional complexes with wild-type monomers or have no effect. In order to discriminate between these possible effects, we assayed the enzymatic activity of a 1∶1 mixture of NME3-P89S with wild-type protein, which would reflect the situation in a *t*/+ male. We combined plasmids encoding the wild-type and the *t*-allele at an equal ratio prior to *in vitro* transcription and translation, purified the complexes, and measured their enzymatic activity. The activity dropped to 58% of that of the wild-type protein, suggesting that both the wild-type and the mutant protein contribute to the total enzyme activity (Figure 3A).

In order to assess whether the *Nme3 t*-allele acts as an antimorph (dominant-negative) or hypomorph (semi-functional) *in vivo* we took a transgenic approach. If the former were the case, a transgenic construct expressing the *t*-allele should enhance the transmission rate of a *t*-haplotype from a *t*/+ male, since a dosage increase of the *t*-allele should further reduce endogenous NME3 activity. In contrast, if the *t*-allele acts as hypomorph the transgene would provide extra NME3 activity to the endogenous gene products and thus the *t*-haplotype transmission should drop.

We created a transgene construct (*Tg(Nme3)H17bgh*, abbreviated *Tg^t^*) expressing the *Nme3^t^* allele in haploid sperm cells using the testis–specific *Ace* promoter (Figure 3B) [20]. Northern blot analysis and quantification showed that the transgene construct was expressed at approximately 80% of the level of the two endogenous alleles from a *t^6^/+* male (Figure 3C). We generated *t^6^/+* mice carrying one wild-type and one *t*-allele, along with *t^6^/+* males additionally expressing the transgene. The former should maintain a 1∶1 ratio of NME3 to NME3-P89S, while the latter should produce around 2.6-fold more NME3-P89S than NME3 (one wild-type allele and one *t*-allele plus approximately 1.6-fold over-expression of the *t*-allele from the *Tg^t^* construct). We determined the transmission rate of *t^6^* from *t^6^*/+;*Tg^t^*/0 and compared it to *t^6^* transmission from control littermates of the genotype *t^6^*/+.

Males (*t^6^*/+) expressing one wild-type and one *t*-allele transmitted the *t^6^*-haplotype to 92.8% of their offspring. An increase of the *t*-allele dosage in hemizygous transgenic males (*t^6^*/+;*Tg^t^*/0) reduced the *t^6^*-transmission to 89% of the offspring (p = 0.02; Table 1), indicating that the *t*-allele does not act as an antimorph, but as a hypomorph.

Biochemical testing confirmed this conclusion. Increasing the amount of NME3-P89S to 2.6-fold of the wild-type protein (1 part NME3: 2.6 parts NME3-P89S), which reflects the relative expression of wild-type and *t*-allele-derived *Nme3* RNA in *t^6^*/+;*Tg^t^*/0 males, significantly increased the enzymatic activity in comparison to the 1∶1 mixture (Figure 3A). Thus, the NME3-P89S protein contributes to the overall enzymatic activity, rather than interfering with activity of the wild-type protein. These biochemical data are consistent with and support the genetic data.

In summary, both data sets identify the *t*-allele of *Nme3* as a hypomorphic allele acting as distorter of *t*-haplotype transmission.

**Figure 4:**
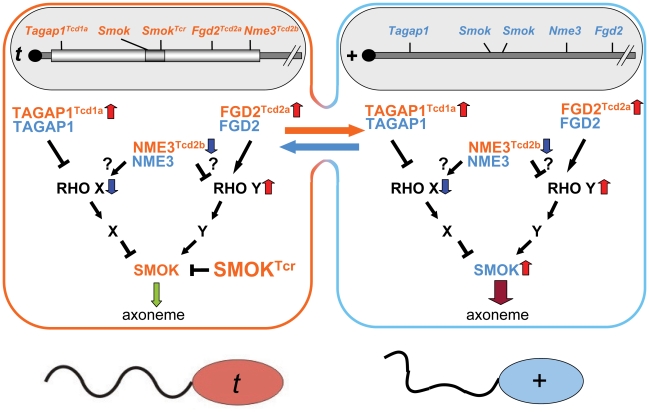
Model of the role of NME3 in *t*-haplotype transmission ratio distortion. *Nme3^Tcd2b^* encodes a hypomorphic allele of *Nme3* leading to reduction of NME3 activity in sperm derived from a *t/+* male. NME3 may be an activator of the inhibitory Rho signaling pathway or an inhibitor of the activating pathway controlling SMOK activity, or both. NME3^Tcd2b^ thus might synergize with TAGAP1^Tcd1a^ to reduce inhibition and/or with FGD2^Tcd2a^ to enhance activation of SMOK in all sperm. The combined activity of all distorters leads to impairment of sperm motility, which is rescued exclusively in *t*-sperm by the responder encoding the dominant-negative variant SMOK^TCR^, resulting in transmission ratio distortion in favor of *t*-sperm. Red upward pointing arrows indicate up-regulation, blue down-pointing arrows down-regulation; the green down-pointing arrow symbolizes rescued, the dark-red down-pointing arrow impaired flagellar motility.

The ability of the mouse *t*-haplotype to promote its transmission from *t*/+ males to a high proportion of their offspring is due to the unusual properties of the responder, *Smok^Tcr^*, a dominant negative protein kinase which is retained in the haploid sperm cells expressing the gene and able to rescue the impairment of sperm motility caused by the distorters [9]. These latter act as QTLs, which additively contribute to the high transmission rate of the responder. We have previously identified two distorters, which act as hypermorphs, the Rho-GAP *Tagap1* and the Rho-GEF *Fgd2* [10], [11]. Although the two proteins have antagonistic effects on Rho activity, excess activity of either gene enhances the transmission rate of the *t*-haplotype from *t*/+ males. Thus, we proposed that *Tagap1* controls a negative regulator and *Fgd2* an activator of SMOK, the wild-type form of the responder SMOK^Tcr^ [11]. Increased down-regulation of the negative regulator by *Tagap1* and increased up-regulation of the activator by *Fgd2* both contribute to hyperactivation of SMOK, leading to impairment of sperm motility. This deleterious effect of the distorters is counterbalanced by SMOK^Tcr^, which exclusively rescues *t*-sperm resulting in TRD (Figure 4).

It is not yet clear how *Nme3* interacts with the Rho signaling cascades involved in TRD. Recent reports have revealed negative interactions between NMEs and Rho small G protein signaling [21]–[23]. *Nme1* can act as a negative regulator of CDC42 by binding to the PH domain of the CDC42-GEF DBL. It can also inhibit CDC42 by direct interaction [21], [22]. Similarly, NME1 has been demonstrated to inhibit RAC1 activity by interacting with the RAC1 activator TIAM1, an effect which is independent of its nucleoside disphosphate kinase activity [23]. It is unknown whether NME3 may function similarly. However, since genetic reduction of *Nme3* promotes TRD, it is likely that the NME3 protein is able to activate the inhibitory pathways controlling SMOK activity. Alternatively, it cannot be excluded that NME3 may inhibit the activating pathway or exert both, activating and inhibiting functions. The former effect might be caused by local increase of the GTP concentration promoting activation of Rho, the latter in a manner similar to that observed for NME1 action on CDC42. In any case, the NME-P89S protein expressed by the *t*-haplotype leads to reduction of total NME3 activity and thus to down-regulation of the inhibitory pathway and/or up-regulation of the activating pathway (Figure 4). The possibility of some other link between NME3 and SMOK, though less likely, cannot be excluded.

Positional mapping of *Nme3* within the *t*-allele places it in the *Tcd2* region, demonstrating for the first time a distorter region that conclusively contains several distorter loci (*Fgd2* and *Nme3*). Accordingly, the two distorters in the *Tcd2* region are termed *Fgd2^Tcd2a^* and *Nme3^Tcd2b^*. A previous report had suggested the presence of two distorters in the *Tcd1* region, which was confirmed by our data, but isolation of the second distorter in the *Tcd1* region has not yet been reported [10], [24], [25].

Various *Nme* genes are expressed in male germ cells, several of them more specifically and prominently than *Nme3* [14], [26]. However, to our knowledge this study is the first providing strong evidence for a role of a group I *Nme* gene in sperm function. It is conceivable that other *Nme* genes as well are involved in the control of sperm motility and affect non-Mendelian inheritance, either as enhancers or suppressors of *t*-haplotype TRD. In this way they may contribute to the high variability of *t*-haplotype transmission observed in different genetic backgrounds [27]. Also, partial redundancy among *Nme* genes may complement the loss of *Nme3* function, since *Nme3* ^−/−^ males show no gross defects in viability and fertility (this report, data not shown).

The human group I gene *NME1* (Nm23-H1) was the first metastasis suppressor gene discovered and has been shown to suppress tumor cell motility in a variety of cancer models [28]. A similar function as an inhibitor of cell motility in a breast cancer cell line has been shown for human NME3 (NM23-H3) [29].

Together with our previous findings implicating Rho signaling in the control of sperm motility, this report provides further evidence for a functional conservation of the signaling networks controlling cell motility in somatic cells and in spermatozoa.

## Materials and Methods

### Ethics Statement

Animal experiments were approved by the ethics committee of the Regierungspräsidium Freiburg (registration number T-00/28) and the LAGeSo Berlin (registration numbers ZH120 and Reg 0248/03).

Primer oligonucleotides are listed in Table S1.

### Transcript Analysis

We amplified *Nme3* transcripts by RT-PCR of the *Nme3* coding region from mouse testis RNA isolated from different wild-type strains and *t*-haplotypes (primers Nme3-s and Nme3-as). We isolated the amplicons (622 bp), cloned them into pBS-SK (Stratagene), and sequenced several independent clones for each genotype. We isolated *Nme3* clones from a *t^6^/t^w5^* testes cDNA library by PCR-screening of subpools and colony hybridization [11] using the *Nme3* cDNA as a probe and sequenced the library clones as above. We performed Northern blot analysis using the NorthernMax-Gly Kit (Ambion) according to the manufacturer's instructions. For *in situ* hybridization analysis, DIG-labelled *in vitro* transcribed antisense RNA corresponding to the coding region of *Nme3* (primers Nme3-s and Nme3-as) was hybridized to 10 µm frozen sections as described [30].

### Gene Targeting and Transgene Constructs

We targeted the *Nme3* locus in CJ7 ES-cells [31] by replacing exon 1 and part of exon 2 (bp 33550 to 33847 in BAC 126c8, accession number AF220294.1) with a *Pgk1*-neo-polyA (Pgk1-neoflox3xpA) - cassette [11] as depicted in Figure 2A. We included a diphtheria toxin cassette for negative selection. To construct the targeting vector, we isolated the left- and right homology arms (4695 bp and 2180 bp, respectively) of the targeting construct by PCR and cloned both arms on either side of the selection cassette, introducing a *Sal*I site for linearization at the end of the left arm. We electroporated CJ7 ES-cells with the linearized targeting construct and selected, isolated, and analyzed clones according to standard procedures [32]. Correctly targeted ES-clones were identified by Southern hybridization of *Xba*I digested genomic DNA with the left (5′) probe (LP) and right (3′) probe (RP) (Primers LP-s and LP-as, 1052 bp probe fragment; RP-s and RP-as, 814 bp probe fragment). Both probes detect a 11.206 kb *Xba*I fragment in wild-type. Upon successful targeting, the LP detects a 7.228 kb fragment and the RP a 5.354 kb fragment. The *Nme3^t^* transgenic construct consists of the angiotensin converting enzyme (*Ace*) spermiogenesis-specific promoter including the transcriptional start site (position −91 to +17) [20] followed by the cDNA of *Nme3* exon 1 to 4, and genomic sequence comprising intron 4/5, exon 5 and 141 bp from the 3′- untranslated region, ending 12 bp upstream of the endogenous polyadenylation (pA) signal, which we replaced by the SV40 pA sequence from pCS2+. We flanked this expression cassette with 2 copies of the chicken beta-globin insulator on both sides [33] (Figure 3B).

### Biochemical Assays

We cloned the *Nme3* alleles into the pET30c vector (Novagen) in-frame with a 6xHis and S-tag. Since expression of full-length recombinant proteins in E. coli was unsuccessful, we used the constructs for *in vitro* transcription/translation reactions (IVT) with the TNT T7 Quick Coupled Transcription/Translation System (Promega) according to the manufacturer's instructions. Plasmid concentrations were kept constant relative to the reaction volumes. We purified recombinant NME3 using Ni-NTA agarose (Qiagen) (12.5 µl bed volume/50 µl IVT reaction). We added 0.6 ml Ni-NTA buffer for binding (50 mM NaH_2_PO_4_, 30 mM NaCl, 20 mM Imidazole, 1× complete protease inhibitors EDTA-free (Roche), pH 8) and incubated at 4°C for 1.5 h with agitation. Samples were then washed 3× in Ni-NTA-buffer (0.8 ml) and NME3-protein was eluted with 45 µl elution buffer (Ni-NTA buffer containing 250 mM imidazole). For quantification of protein, we loaded 20 µl of eluted protein on a NuPAGE 4–12% Bis-Tris Gel (Invitrogen) and blotted with the I-blot system (Invitrogen) on a PVDF membrane (Millipore). After blocking 1–2 h at RT using 5% skim milk powder in TBS-T/0.2% Tween, we probed the blot with an anti-S-tag antibody (1∶500 dilution, Delta Biolabs) overnight at 4°C, followed by incubation with donkey anti-rabbit HRP-coupled secondary antibody (1∶10000 diluted, Jackson Immunoresearch), and detected the signal with the ECL Advance Western Blotting Detection Kit from GE Healthcare. Densitometry quantification of Western blots was performed using ImageJ software.

We analyzed NME3 protein preparations for nucleoside diphosphate kinase activity using a transphosphorylation assay followed by thin layer chromatography (TLC) essentially as described [34]. We used 20 µl NME3 protein eluate in a reaction mixture containing 10 mM HEPES, 20 mM NaCl and 2 mM MgCl_2_, 2 mM ATP, 1 mM TDP and 2 mM [γ-^32^P] ATP. We took 6 µl samples after each 15, 30, and 45 minutes incubation at room temperature and stopped the reaction with 1 µl of 50 mM EDTA (pH 8.0). As a positive control we used 0.1 U of nucleoside 5′-diphosphate kinase from bakers yeast (Sigma Aldrich N0379). Reactions were analyzed by TLC on PEI-cellulose plates (Macherey-Nagel) using 0.75 M KH_2_PO_4_ (pH 3.65) as running buffer. We exposed dried TLC plates to phosphorimager screens and quantified with ImageJ. Wild-type NME3 and NME3-P89S protein activities were normalized to total protein levels.

## Supporting Information

Figure S1 *Nme3* maps to the *Tcd2* region of the *t*-haplotype. (A) Genetic maps of the wild-type *t*-complex (+) and the *t*-haplotype (*t*). The proximal partial *t*-haplotype *t^w18^* arose by recombination between a *t*-haplotye and a wild-type chromosome within inversion 4 (In(17)4) resulting in loss of the distorter *Tcd2*. (B) Genomic Southern blot analysis using the coding sequence of *Nme3* as probe demonstrates the presence of a non-polymorphic *Nme3* fragment in *t^w18^*, as shown by the presence of a 2.19 kb *Bgl*I fragment (+, *t*), and of a 1.43 kb knock-out allele fragment in *t^w18^*/+; *Nme3* ^+/−^ genomic DNA. Since *Nme3* is located distal to *Fgd2* in the *t*-haplotype and *t^w18^* carries the wild-type allele of *Fgd2* (Figure 1a in [11]) we conclude that the 2.19 kb fragment in *t^w18^* represents the wild-type *Nme3* allele. In addition, we did not detect *t*-allele *Nme3* transcripts in *Nme3^tm5Bgh^*/+;*t^w18^*/+ testes-derived cDNA clones, nor *t*-allele derived amplicons in *t^w18^* genomic DNA (not shown).
(TIF)

Table S1 Primer sequences for transcript and genomic analysis, generation of transgenic and gene targeting constructs, and genotyping.
(DOC)
