# Supplementary figures and images for: The Nucleoside Diphosphate Kinase Gene Nme3 Acts as Quantitative Trait Locus Promoting Non-Mendelian Inheritance
Source: PLoS Genet. 2012 Mar 15;8(3):e1002567. doi: 10.1371/journal.pgen.1002567 (PMC3305403; doi:10.1371/journal.pgen.1002567)

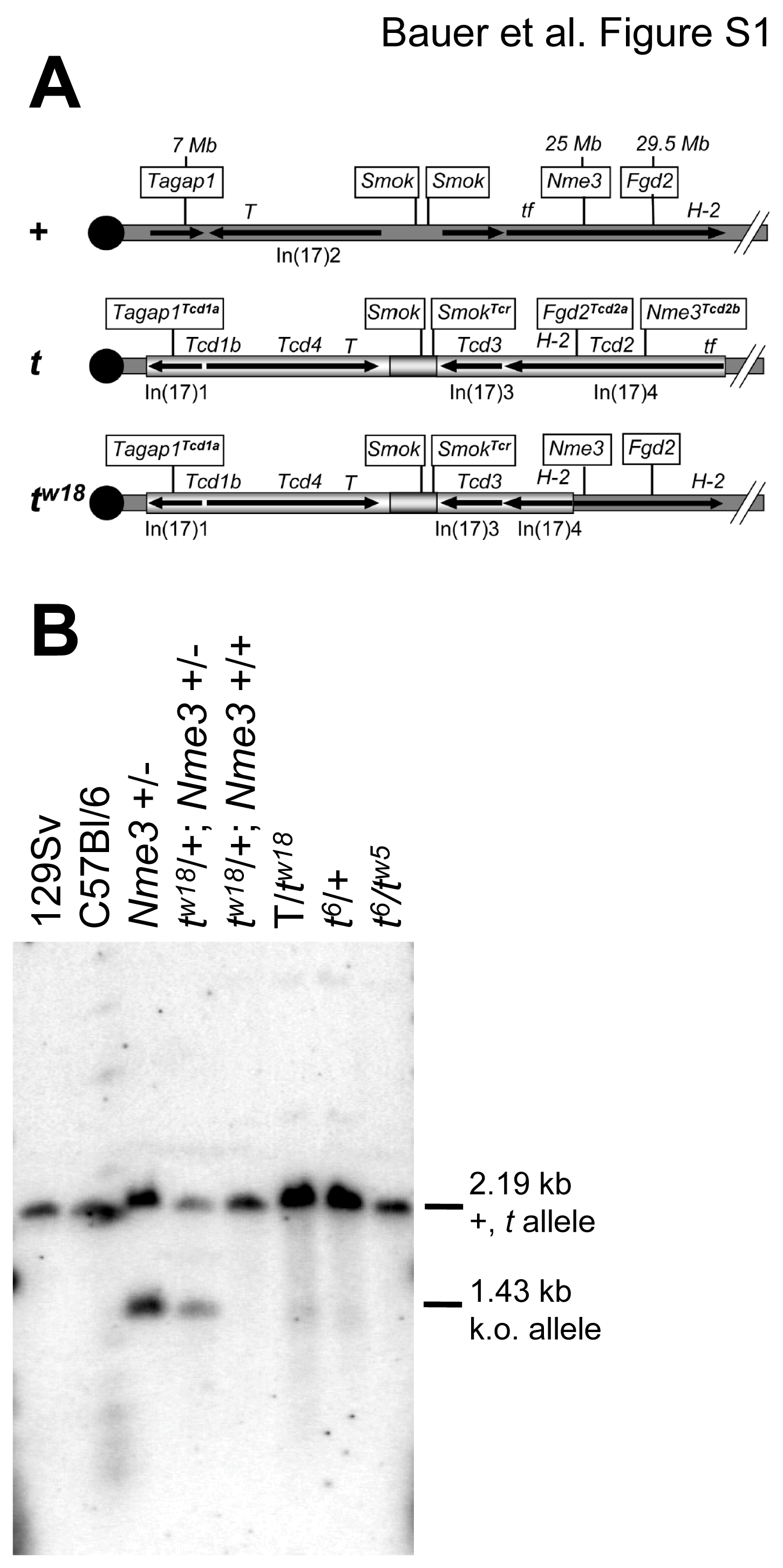

Supplement: Figure S1 — Nme3 maps to the Tcd2 region of the t-haplotype. (A) Genetic maps of the wild-type t-complex (+) and the t-haplotype (t). The proximal partial t-haplotype tw18 arose by recombination between a t-haplotye and a wild-type chromosome within inversion 4 (In(17)4) resulting in loss of the distorter Tcd2. (B) Genomic Southern blot analysis using the coding sequence of Nme3 as probe demonstrates the presence of a non-polymorphic Nme3 fragment in tw18, as shown by the presence of a 2.19 kb BglI fragment (+, t), and of a 1.43 kb knock-out allele fragment in tw18/+; Nme3 +/− genomic DNA. Since Nme3 is located distal to Fgd2 in the t-haplotype and tw18 carries the wild-type allele of Fgd2 (Figure 1a in [11]) we conclude that the 2.19 kb fragment in tw18 represents the wild-type Nme3 allele. In addition, we did not detect t-allele Nme3 transcripts in Nme3tm5Bgh/+;tw18/+ testes-derived cDNA clones, nor t-allele derived amplicons in tw18 genomic DNA (not shown). (TIF) [file pgen.1002567.s001.tif]
