# Supplementary material for: The Nucleoside Diphosphate Kinase Gene Nme3 Acts as Quantitative Trait Locus Promoting Non-Mendelian Inheritance
Source: PLoS Genet. 2012 Mar 15;8(3):e1002567. doi: 10.1371/journal.pgen.1002567 (PMC3305403; doi:10.1371/journal.pgen.1002567)
Supplement: Table S1 — Primer sequences for transcript and genomic analysis, generation of transgenic and gene targeting constructs, and genotyping. (DOC) [file pgen.1002567.s002.doc]

**Table S1**

| **Experiment** | **Primer sequence (5´- 3´)** | **Name** | **Number of cycles/ annealing temp.** | **Product size** |
| --- | --- | --- | --- | --- |
| **Transcript and genomic analyses** | | | | |
| **Amplification of *Nme3* cDNA and genomic for seq. analysis, semi-quant. PCR*, RFLP analysis tw18, Northern- and *in situ* hybridization probe generation** | Atgatctgtctggtgct  cgactaggttgggttga | Nme3-s  Nme3-as | 20*****, 25*****, 28, 30*****, 35* cycles  53 °C | cDNA: 622 bp  genomic: 1199bp |
| **Expression analysis (beta actin control)** | TGGAATCCTGTGGCATCCATGAAA  taaaacgcagctcagtaacagtccg | Act-s  Act-as | 25 cycles/ 58 °C | 349 bp in  NM_007393 |
| **Generation of a Gapdh-probe** | GGTGCTGAGTATGTCGTGGA  CACATTGGGGGTAGGAACAC | Gapdh-s  Gapdh-s | 25 cycles/ 55 °C | 450 bp |
| **Gene targeting** | | | | |
| **Amplification of left arm of *Nme3***  **targeting construct (Fig. 2A)** | ACACCTGGTGCCGAGAG  tttcctcccttgtcctgcggtaca | LAs SalI  LAas | 25 cyles/ 52 °C | 4695 bp |
| **Amplification of right arm of *Nme3***  **targeting construct (Fig. 2A)** | Gatcgtgcgtcgctttg  GGGCAGCCAGGTTCGTC | RAs-BamHI  RAas-XbaI | 28 cyles/ 54 °C | 2180 bp |
| **Left (5´-) probe for detection of *Nme3***  **homologous recombination (Fig. 2B)** | Tagctgtagccctccctagttt  cccagcttgttatgcag | LP-s  LP-as | 28 cyles/ 54 °C | 1052 bp |
| **Right (3´-) probe for detection of *Nme3***  **homologous recombination (Fig. 2B)** | GGGCCAACCTCTAGTCT  CCTTCGTGCGCATCACA | RPs  RPas | 25 cyles/ 54 °C | 814 bp |
| **Genotyping for *Nme3* wild-type (wt) or knock-out allele (mut) by PCR** | GGTATGGAGCAGAGCAGCAG  CGGGAGTACTGGCTTCAGG | Ndk3wtS  Ndk3wtas | 38 cyles/ 61 °C | 300bp wt band |
|  | Caaagggcctacccgcttcca  GGAGGAGTAGCGACCTATACCAG | Ndk3mutAS  Ndk3mutAS | 38 cyles/ 59 °C | 360bp mut band |
| **Transgenic construct** | | | | |
| **Amplification of ACE promoter** | AGGGCCCTTGGGGTCAGG  ggccgcaggaaagcagag | AceP-s  AceP-as | 35 cycles/ 56 °C | 109 bp |
| **Genotyping for transgenic *Nme3***  **construct** | CGGCCTCACGGAACCAA  TACAGCGGCGTGAACGA | Tg-s  Tg-as | 35 cycles/54 °C | wt band: 656 bp  tg-band: 488 bp |
| **t-haplotype genotyping** | | | | |
| **Genotyping for proximal *t*-haplotype region (Table 1)** | TCATGGACCAACACAAGCTC  CACAAAACTGAAATCTCCCTCTC | Vil-s  Vil-as | 40 cycles/ 57 °C | *t*-band: 195 bp  wt band: 228 bp |
| **Genotyping for distal *t*-haplotype region**  **(marker Hba-4ps)** | GAGTGACCTGCATGCCCACAAGCTGTG  GACCTGTGGAGACAGGAAGGGTCAGTG | HB1  HB2 | 35 cycles/ 66 °C | *t*-band: 220 bp  wt band: 201 bp |
|  |  |  |  |  |
